# The Role of Infection in Acute Exacerbation of Idiopathic Pulmonary Fibrosis

**DOI:** 10.1155/2019/5160694

**Published:** 2019-01-03

**Authors:** Dong Weng, Xian-Qiu Chen, Hui Qiu, Yuan Zhang, Qiu-Hong Li, Meng-Meng Zhao, Qin Wu, Tao Chen, Yang Hu, Liu-Sheng Wang, Ya-Ru Wei, Yu-Kui Du, Shan-Shan Chen, Ying Zhou, Fen Zhang, Li Shen, Yi-Liang Su, Martin Kolb, Hui-Ping Li

**Affiliations:** ^1^Department of Respiratory Medicine, Shanghai Pulmonary Hospital, Tongji University, School of Medicine, Shanghai, China; ^2^School of Medicine, Suzhou University, Suzhou, China; ^3^McMaster University Hamilton, Department of Medicine and Pathology/Molecular Medicine, ON, Canada

## Abstract

**Background:**

Acute exacerbation of IPF (AE-IPF) is associated with high mortality. We studied changes in pathogen involvement during AE-IPF and explored a possible role of infection in AE-IPF.

**Objectives:**

Our purpose is to investigate the role of infection in AE-IPF.

**Methods:**

Overall, we recruited 170 IPF patients (48 AE-IPF, 122 stable) and 70 controls at Shanghai Pulmonary Hospital. Specific IgM against microbial pathogens and pathogens in sputum were assessed. RNA sequences of pathogens in nasopharyngeal swab of IPF patients were detected by PathChip. A panel of serum parameters reflecting immune function were assessed.

**Results:**

Antiviral/bacterial IgM was higher in IPF vs. controls and in AE-IPF vs. stable IPF. Thirty-eight different bacterial strains were detected in IPF patient sputum. Bacteria-positive results were found in 9/48 (18.8%) of AE-IPF and in 26/122 (21.3%) stable IPF. Fifty-seven different viruses were detected in nasopharyngeal swabs of IPF patients. Virus-positive nasopharyngeal swabs were found in 18/30 (60%) of tested AE-IPF and in 13/30 (43.3%) of stable IPF. AE-IPF showed increased inflammatory cytokines (IL-6, IFN-*γ*, MIG, IL-17, and IL-9) vs. stable IPF and controls. Mortality of AE-IPF in one year (39.5%) was higher compared to stable IPF (28.7%)*.Conclusions*. IPF patients had different colonization with pathogens in sputum and nasopharyngeal swabs; they also displayed abnormally activated immune response, which was exacerbated during AE-IPF.

## 1. Background

Idiopathic pulmonary fibrosis (IPF) is a fatal disease with unknown etiology, characterized by a radiographic and pathologic pattern of usual interstitial pneumonia (UIP). Patients with IPF have a median survival time of 3 years [1], following either a progressive course of worsening respiratory function, or a more rapid decline described as acute exacerbation (AE-IPF) [2]. The common criteria for AE-IPF include a diagnosis of IPF with acute worsening of dyspnea in the preceding month, new radiographic opacities or ground glass on computed tomography (CT), and exclusion of alternative causes (e.g., infection, congestive heart failure, and pulmonary embolism). AE-IPF is a dangerous condition with a high mortality (often >50%) [3–8]. In some reports, the one-year mortality of patients with AE-IPF was almost 100% [4, 5].

The previously used definition of AE-IPF [7] has proven to be unclear, and a consensus on the definition even in expert centers has been difficult [9]. Recently, an international working group had suggested that it would be important to rule out obvious infections for IPF patients with deteriorating symptoms over less than one month but had also acknowledged that AE following a triggering event might end as bad as idiopathic AE episodes [10]. Known triggers for AE-IPF include lung surgery, bronchoscopy with BAL, aspiration, pollution, and preceding infections, but the exact causes and mechanisms for AE-IPF remain unclear [3]. The fundamental to the concept of idiopathic AE-IPF is the assumption that these episodes are a distinct clinical entity and that it is important to distinguish them from acute respiratory deterioration of known cause, which was questioned by more recent data [9, 11]. Many publications do not report sufficient details to confidently exclude infections in events reported as AE-IPF. Indeed, it is almost impossible to exclude a lot of different infections, especially viral pathogens in the clinical situation due to unreliable commercial tests and inconsistent clinical testing. In our clinical observations, many patients of IPF with AE experienced common cold symptoms before their rapid decline. Typical common cold symptoms include cough, worsening dyspnea and mild fever—all of them have been included in the description of AE-IPF events [11]. Further, recent evidence points towards a potential role of an altered lung microbiome in triggering IPF progression, including AE-IPF [12–17].

In this study, we asked whether IPF patients may be particularly sensitive to common infectious triggers, some of them then developing AE-IPF. We aimed to investigate the association of AE-IPF with microbial colonization and/or latent infection.

## 2. Methods

### 2.1. Subjects and Selection Criteria

**Figure 1:**
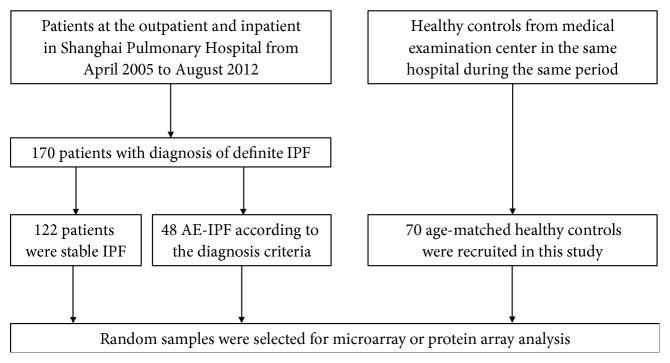
Flow chart of patient enrollment.

We prospectively enrolled 170 definite IPF patients (122 stable IPF without corticosteroid, immunosuppression, and antibiotic therapy and 48 AE-IPF) who were managed at the Shanghai Pulmonary Hospital between April 2005 and August 2012, either in the outpatient or inpatient department (Figure 1). The diagnosis of IPF was established according to international guidelines [6]. All patients were treated according to the IPF guidelines [6] after admission. The therapies for AE-IPF in our group were as follows: oxygen therapy (including nasal catheter, mask oxygen inhalation, and noninvasive ventilation when necessary); glucocorticoids (mainly methylprednisolone 80-320 mg/day); antibiotics (mainly broad-spectrum antibiotics, empirically covering common pathogens of respiratory tract infections, antifungal therapies for patients accompanied with fungal infection); antioxidation therapy (glutathione); immune support therapy (gamma globulin, etc.); nutritional support; and symptomatic therapy. Regarding episodes of AE-IPF [6–8], we applied the following criteria of AE-IPF: (1) established diagnosis of definite IPF; (2) progressive dyspnea within 1 month, and presented with hypoxemia at room air; (3) new bilateral radiographic opacities in CT scan; (4) absence of identifiable etiology including pulmonary embolism, pneumothorax, congestive heart failure, and pneumonia (using usual clinical criteria with presence of fever > 38.5°C, elevated WBC count > 15,000, and pulmonary infiltrates resolving with antibiotic therapies). We also recruited 70 healthy age-matched controls from the medical examination center of the same hospital during the same time period (April 2005 and August 2012). Healthy controls were admitted to our hospital for health examination and did not get diagnosed with any diseases, showing normal lung function and chest X-ray. All of the subjects in the study were followed up by telephone or outpatient appointments until Sept. 2014. Clinical data of patients and healthy controls were prospectively collected and analyzed. The ethics committee of the College of Medicine and Life Science, Tongji University, approved this study. All participants gave written informed consent.

Fasting venous blood specimens were sampled from IPF patients and healthy controls on the day after admission or on the day of medical examination. All IPF patients underwent pulmonary function tests before acute exacerbation. Arterial blood was sampled from IPF patients (at room air) on the day of admission to perform arterial blood gas analysis.

The severity of pulmonary fibrosis and lesion involvement was assessed according to the Helbich quantified HRCT scoring system [18], and ground-glass opacities (including consolidation), reticular shadow, and honeycombing were included as indicators for fibrosis assessment. HRCT scans at three levels (aortic arch, tracheal bifurcation, and at a dimension of 1 cm above the diaphragm) were used for HRCT scoring. The total HRCT score was calculated by summing the scores of all levels. A higher HRCT score indicated more severe pulmonary fibrosis (Table 1).

### 2.2. Serum IgM Antibody Detection

Peripheral blood was centrifuged in a procoagulant tube at 3000 rpm for 15 min, and serum was separated for determination of pathogen-specific IgM. The immunofluorescence-based Pneumoslide IgM kit (Vircell, Spain) was used to detect specific IgM antibody against *Legionella*, *Mycoplasma pneumoniae*, *Q fever Rickettsia*, *Chlamydia pneumoniae*, *Adenovirus*, *respiratory syncytial virus*, *influenza A virus*, *influenza B virus*, *and parainfluenza virus.* The cytomegalovirus (CVM) IgM ELISA kit (BioAssay, USA) was used to detect the IgM antibody response to CMV.

### 2.3. Culture of Pathogens in Sputum

Spontaneous sputum samples were collected from all IPF patients in the early morning for 3 consecutive days after hospital admission, and samples were immediately (within 30 min) sent for sputum culturing. Qualified samples needed to meet the following criteria: (1) squamous cells < 10 cells/low magnification; (2) white blood cells (WBC) > 25 cells/low magnification; or (3) the squamous cell: WBC ratio < 1 : 2.5. Consistent results obtained more than twice of each sputum culture from the same patient were considered as a single result. If the results of the same patient were inconsistent, sputum cultures were performed again.

### 2.4. Viral RNA Extraction and PathoChip

Random nasopharyngeal swab samples were selected with the help of computational random number generators. RNA was extracted from nasopharyngeal swab stored in virus sampling tube using QIAamp Viral RNA Mini Kits (Qiagen Inc., Hilden, Germany: cat. no. 52906) according to the manufacturer's instructions. The RNA was stored at -80°C. The PathChip (PathGEN Dx, Singapore) can detect over 70,000 full-genome RNA sequences of pathogens (including over 50,000 viruses and 20,000 bacteria) clinically relevant to humans [19]. The RNA samples were purified, fragmented, labeled, and hybridized onto the PathChip according to the manufacturer's protocol. After hybridization, the PathChip was washed, stained, and scanned using the Affymetrix GeneChip system. The Affymetrix image file (.CEL) containing all the raw signal intensities for each PathChip was uploaded into the GIS proprietary software described previously [19], which automatically detects pathogen recognition signatures.

### 2.5. Serum Inflammatory Cytokines Profiling

The first step, soluble proteins from the serum samples of healthy controls (*n* = 6), stable IPF (*n* = 6), and AE-IPF (*n* = 6) were profiled using the RayBio® L-Series 507 Biotin Label-based Antibody Array system (RayBiotech, GA, USA). The next step, we designed a new array (RayBiotech QAH-CUST, RayBiotech Inc.) by using 30 different cytokines selected according to our observations and literature reports, and 60 samples (AE-IPF patients, *n* = 20; stable IPF, *n* = 20; health control, *n* = 20) were examined. The array was used by following the manufacturer's instructions to measure the following cytokines: BMP-7, EOT, Flt-3L, G-CSF, GM-CSF, ICAM-1, IFN-*γ*, IL-10, IL-12 p40, IL-12 p70, IL-13, IL-15, IL-17, IL-1*α*, IL-1*β*, IL-22, IL-4, IL-6, IL-7, IL-9, leptin, LIGHT, MCP-1, MCP-2, MIG, MMP-7, NT-3, PDGF-BB, TGF-*β*1, and TSLP. After developing, slides were scanned, and the images were processed and quantified using Axon GenePix4400A microarray scanners. Intensity was normalized to internal positive controls for comparison.

### 2.6. Statistical Analysis

Statistical analyses were performed using the software program SPSS 13.0. Counting data are presented as mean ± SD. The independent sample Student's *t*-test was used for comparisons between two groups, and one-way ANOVA followed by Tukey's post hoc test was used to compare three groups. The chi-square test was used for constituent ratio comparisons. Pearson's method was used for association analyses. Survival rates were estimated using Kaplan-Meier methods and group comparisons were tested using log-rank test. Statistical significance was defined at an alpha value of *p* < 0.05.

## 3. Results

### 3.1. Demographic Data

**Figure 2:**
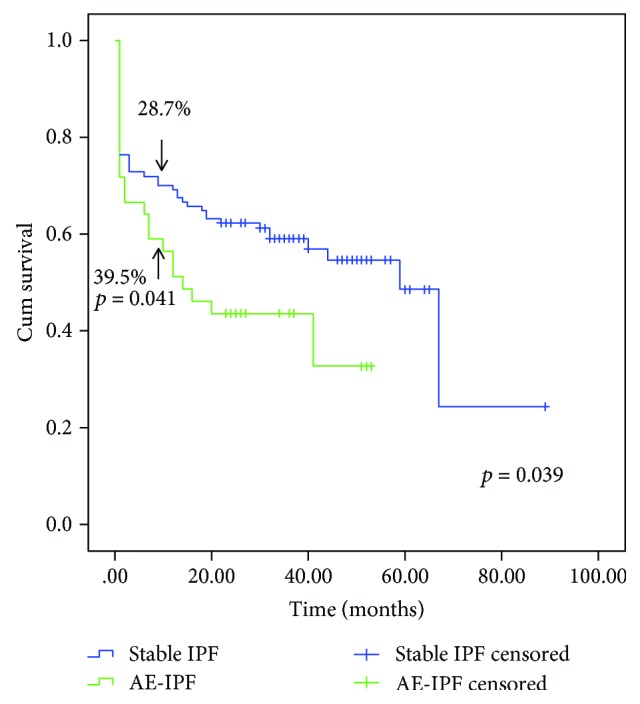
Kaplan-Meier survival analysis showing significantly higher mortality of AE-IPF than IPF (stable IPF) by using the log-rank test (*p* = 0.039). AE-IPF patients (39.5%) had higher one-year mortality than stable IPF (28.7%) (*p* = 0.041). In AE-IPF group (23 cases of death), 19 cases died of respiratory failure by acute exacerbation; 4 died of severe pneumonia. In stable IPF group (50 cases of death), 15 died of acute exacerbation in the period of follow-up, 10 died of pneumonia, 5 died of lung cancer, 2 died of liver cancer, 2 died of intestine cancer, 5 died of myocardial infarction, 2 died of gastrointestinal bleeding, 4 died of pulmonary embolism, 3 died of stroke, 1 died of diabetes, and 1 died of trauma.

A total of 170 patients with IPF were enrolled from Shanghai Pulmonary Hospital. Clinical characteristics of the patients are summarized in Table 2. Controls were age matched, compared with IPF patients, and had less exposure to potential environmental triggers, cigarette smoking, and also no history of recent common cold symptoms. Patients with AE-IPF showed a significantly higher incidence (89.6%) of recent cold symptoms (including more cough, nasal obstruction, rhinorrhea, sore throat, chills, and headache 1-2 weeks before acute exacerbation) compared with stable IPF patients. Patients were followed from the time of the enrollment visit at our hospital until Sept. 2014, and survival analysis was performed. As expected, overall Kaplan-Meier survival analysis showed significantly higher mortality of AE-IPF (23/48, 47.9%) compared with stable IPF (50/122, 41.0%; log rank to compare these curves *p* = 0.039). In AE-IPF patients, 19 patients (19/48, 39.58%) died within one year after the AE episode; of them, 57.9% (11 of 19) died within one month. In stable IPF patients, the 1-year mortality was 28.7% (35/122), which was significantly lower than that of AE-IPF (Figure 2, *p* = 0.041). All deaths occurred within the follow-up period (Sept. 2014).

IPF patients had significantly higher total WBC counts and neutrophils than healthy controls (stable IPF 8.06 ± 2.45, AE-IPF 9.01 ± 4.61, control 6.01 ± 1.46; difference IPF to control *p* = 0.002). AE-IPF patients showed a slightly higher neutrophil percentage than stable IPF, which could indicate latent infections in patients developing AE-IPF. Compared with stable IPF, AE-IPF patients had significantly lower arterial oxygen pressure (*p* = 0.001) and saturation (*p* < 0.0001), while pulmonary function tests indicated that AE-IPF patients had worse FVC (% pred) (*p* = 0.02), worse FEV1 (% pred) (*p* = 0.007), and increased FEV1/FVC ratio (*p* = 0.01). The HRCT scores of AE-IPF patients were significantly higher than that of stable IPF patients (*p* = 0.001).

### 3.2. Antimicrobial IgM, Sputum, and Nasopharyngeal Swap Analysis

#### 3.2.1. Serum IgM Antibodies

Detection of IgM antibody against 10 common pathogens revealed that mycoplasma was the pathogen with the highest IgM positive rate (12.2%) in the serum of AE-IPF patients, followed by legionella (7.3%), adenovirus (7.3%), and RSV (4.9%). In the serum of stable IPF patients, mycoplasma also showed the highest IgM positive rate (5.6%), followed by legionella (4.6%), RSV (3.7%), and influenza B viruses (2.8%). For healthy controls, only mycoplasma (7.1%), adenovirus (2.9%), and parainfluenza virus (1.4%) were positive (Table 3). The total positive rate of IgM antibodies was 36.6%, 19.4%, and 11.4%, respectively, in AE-IPF, stable IPF, and healthy controls (Table 3). The positive rate of IgM in AE-IPF was significantly higher than that seen in both stable IPF (*p* = 0.0290) and healthy controls (*p* = 0.0016), and a nonsignificant difference in the positive rates of IgM was seen when comparing stable IPF and healthy controls.

#### 3.2.2. Culture of Pathogens in Sputum

The categories and constituent ratio of pathogens detected in the sputum of 170 IPF cases are listed in Table 4. A total of 38 bacterial strains were detected. Most prominent were Gram-negative bacteria (89.5%); Gram-positive strains only accounted for 10.5%. In terms of the Gram-negative strains, *Klebsiella pneumonia* was most abundant (26.3%), followed by *Mycobacterium tuberculosis* (21.1%), and *Acinetobacter baumannii* (10.5%). The total detection rates in AE-IPF and stable IPF patients were 18.8% and 21.3%, respectively (Table 4). There was no significant difference between AE-IPF and stable IPF.

#### 3.2.3. Detection of Viruses in Nasopharyngeal Swab

The categories and constituent ratio of viruses detected by PathGEN® PathChip Kit in the throat swab of 30 stable IPF and 30 AE-IPF cases are listed in Table 5. A total of 57 different viruses (40 in AE-IPF and 17 in stable IPF) were detected. Virus-positive rates in AE-IPF patients were 60.0% (18/30) and 43.3% (13/30) in stable IPF. There was no significant difference between AE-IPF and stable IPF (*p* = 0.1965). In the AE-IPF group, HHV (human herpesvirus) and INF A (influenza virus A) accounted for 37.5% (15/40) and 30% (12/40), respectively. In stable IPF patients, HHV and HRV (human rhinovirus) were both accounted for 23.5%.

#### 3.2.4. Inflammatory Cytokines

**Figure 3:**
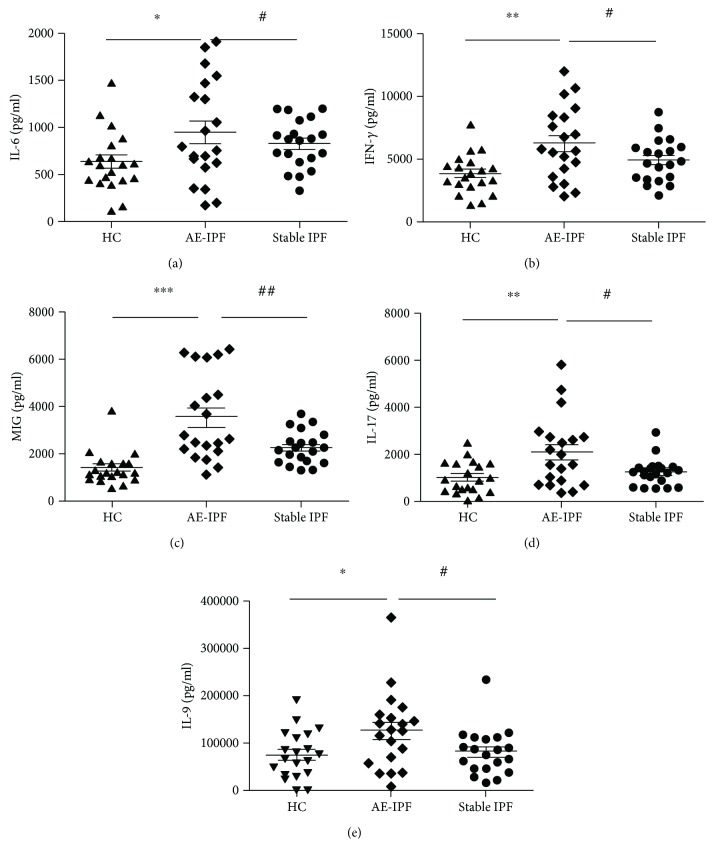
Antimicrobial inflammatory cytokines in AE-IPF. Serum of 40 IPF patients (20 AE-IPF and 20 stable IPF) and 20 controls was examined with protein microarray analysis. When compared with controls and stable IPF patients, AE-IPF patients showed significant increases in the levels of IL-6, IFN-*γ*, MIG, IL-17, and IL-9. One-way ANOVA followed by Tukey's post hoc test was used to compare three groups. ^∗^*p* < 0.05, ^∗∗^*p* < 0.01, ^∗∗∗^*p* < 0.001 AE IPF vs. controls. ^#^*p* < 0.05, ^##^*p* < 0.01, ^###^*p* < 0.001 AE IPF vs. stable IPF.

To investigate the association between AE-IPF and infections, we detected the expression levels of antimicrobial inflammatory cytokines in the serum of randomly selected healthy controls (*n* = 20), stable IPF (*n* = 20), and AE-IPF patients (*n* = 20) using RayBiotech QAH-CUST microarray. When compared with controls and stable IPF patients, AE-IPF patients showed significant increases in the levels of IL-6, IFN-*γ*, MIG, IL-17, and IL-9 (Figures 3(a) and 3(e)). These cytokines had critical roles in anti-infection responses. These results suggested infections might play a role in the initiation and development of AE-IPF.

## 4. Discussion

IPF is a disease of unknown etiology that is usually progressive, either in a linear or stepwise manner [1]. In this study, the patients were all diagnosed definite IPF with UIP pattern in HRCT prior to acute exacerbation. There are no changes in diagnostic criteria of definite IPF between 2011 [6] and 2018 [20] guidelines. AE-IPF is an episode of exacerbation that develops rapidly within a few weeks and often occurs unexpectedly during the stable phase of IPF. All of the patients were treated according to the IPF guidelines [6] after admission (data not shown). AE-IPF is associated with high mortality and poor prognosis [3]. The one-year mortality is variable between 40–100% [4]. In our study, AE-IPF patients had a one-year mortality of almost 40% after the episode; 58% (11 of 19) died within the first month. Moua et al. just published a large cohort of patients with IPF and other fibrotic lung disease from the United States who were admitted to the hospital with acute respiratory worsening, having a one-year mortality of almost 80% [5]. The cohort in the report of Moua et al. had an average lower FVC (62% pred.) than our stable IPF group (FVC 84% pred.), but their average FVC was similar to our AE-IPF group (FVC 59% pred.). This could be explained by differences in the study population (ours was only IPF) and also differences in the health care systems (hospitalizations for diagnostic purposes and milder respiratory problems are much more common in China compared to the US). While we do not have follow-up lung function data from all AE-IPF patients except the information about their death, our follow-up data of stable IPF patients show a one-year decline in FVC (270 ± 206 ml) similar to recent studies [21, 22]. The stable IPF patients in our study showed a relatively high mortality (50/122, 41.0%) during follow-up; half of them (25/50) died due to nonrespiratory reasons (different cancers, myocardial infarction, gastrointestinal bleeding, pulmonary embolism, stroke, and others), 15/50 died due to AE in the period of follow-up, and 10/50 died of pneumonia. In stable IPF patients, the one-year mortality was 28.7% (35/122), which was significantly lower than that of AE-IPF (*p* = 0.041).

The earlier definition of AE-IPF warranted exclusion of acute infections of the lower respiratory tract, ideally by tracheobronchial aspirate or bronchoscopy. This is not always and consistently done across centers, not even within the rigid setting of clinical trials in which AE-IPF were used as endpoint [22]. Our clinical observations suggest that rapid worsening or AE of IPF are often preceded by “cold-like” symptoms without clearly establishing an acute infectious respiratory disease. Recently, several studies suggested that progression of IPF seems to be associated with bacterial or viral infection and/or abnormal composition of the lung microbiome, which is a good reason to revisit the definition of AE-IPF [3, 12–17]. Afarwal and Jindal [4] have shown that infections are able to induce an inflammatory cascade in the lung of IPF patients, thus leading to a rapid deterioration of stable IPF. The recent study by Moua and colleagues suggested that the prognosis of AE-IPF is equally poor, regardless of them being idiopathic or triggered by infections [5]. These issues were addressed by an international working group of IPF experts proposing that AE can be triggered events (through occult or detectable infections, lung surgery, or aspiration) or can be idiopathic with similar outcomes [10]. While true pneumonias should not be termed AE according to this document, it is acknowledged that definite exclusion of potential triggers is no longer required for the definition of an AE-IPF episode.

In our study, we basically used the AE-IPF definition proposed in the 2016 document [10], even if we started to enroll subject ten years earlier. We aimed to investigate whether abnormal bacterial, viral, or antimicrobial immune responses were related to the development of AE-IPF. Our data show that AE-IPF patients had significantly higher rates of antimicrobial IgM in their serum compared to stable IPF and significantly more neutrophils. This indicates that infectious pathogens, particularly cold-causing viral and bacterial pathogens, might be an important triggering factor in a substantial number of AE-IPF. Similar findings have been reported before in smaller studies [23]. We also investigated virus sequences in nasopharyngeal swabs of IPF patients. Our data (Table 5) showed that virus-positive samples were highest in the AE-IPF group (60.0%) but also considerable in stable IPF (43.3%). In the AE-IPF group, human herpesvirus and influenza A were the most prominent viruses. Of note, it has been shown before that herpes virus is associated with IPF [24] and can cause exacerbation of pulmonary fibrosis in animal models [25, 26]. Further, acute exacerbation of IPF has been reported after influenza A vaccination [27]. The presence of influenza A in AE-IPF patients supports the notion that cold-associated infections are able to trigger these events. In contrast, Wootton et al did not find evidence for viruses in AE of IPF in a rather large study where they used bronchoscopies and molecular analysis for viral particles [28]. However, even with such a sophisticated approach, it is not possible to rule out a relationship between acute viral infection and subsequent development of AE-IPF because the time between the onset of symptoms and the sampling of bronchial fluid may be too long to detect viral particles [29]. In our study, we also found significantly increased inflammatory cytokines in AE-IPF patients that may indicate a biological process against an infection. IL-6, IFN-*γ*, MIG, IL-17, and IL-9 are important inflammatory cytokines in the anti-infection immune response [30–44] and were all increased in the blood of patients with AE-IPF in our cohort. The changes of cytokines mainly reflect the inflammation cascade amplification effect of AE-IPF. Pathogen infection might be a trigger or a secondary infection in later stage. This also provides supporting evidence for glucocorticoid therapy in AE-IPF. Our data also shows that the antimicrobial IgM serum response against respiratory pathogens and positive sputum cultures tended to be higher in stable IPF patients compared to healthy subjects, suggesting a certain level of latent infection in stable IPF patients. Positive rate of MTB in our study was relatively high. This might be in keeping with the local prevalence. MTB was a long-term infection, while other pathogens might be removed by effective therapies in short time. This meant the detective rate of MTB might be higher than other pathogens in sputum. However, it may be an area of further interest for future work.

Our study has several limitations. The data is observational and even with the significant increase in the anti-infectious cytokines, we have no clear evidence to support that the infections caused AE-IPF through this immune response. However, our group had reported the relationship between virus infection and AE-IPF using animal model [26] recently. The single-center design is always somewhat problematic as certain findings may be influenced by local practice and also local differences in the incidence and prevalence of certain infections. Further, our study would be stronger if we had an independent cohort to validate the finding. Nevertheless, this is one of the largest studies of AE-IPF where the role of infections and anti-infection immune response was explored with advanced diagnostic tools.

## 5. Conclusion

Taken together, our data suggest that latent infections and/or abnormal colonization in the lungs of IPF patients and “common cold”-associated virus infections together with a significantly elevated anti-infection immune response might be a common triggering factor for AE-IPF.

## Figures and Tables

**Table 1:** HRCT scoring criteria.

HRCT imaging	Scores
Ground-glass changes	
No ground-glass opacities	0
Ground-glass opacity area < 5%	1
Ground-glass opacity area 6-24%	2
Ground-glass opacity area 25-49%	3
Ground-glass opacity area 50-74%	4
Ground glass opacity area > 75%	5
Reticular changes (septal thickening)	
No reticular shadow	0
Reticular shadow area < 5%	1
Reticular shadow area 6-24%	2
Reticular shadow area 25-49%	3
Reticular shadow area 50-74%	4
Reticular shadow area > 75%	5
Honeycomb-like changes	
No honeycombing	0
Honeycombing area < 5%	1
Honeycombing area 6-24%	2
Honeycombing area 25-49%	3
Honeycombing area 50-74%	4
Honeycombing area > 75%	5
Total	

The “area” indicates the percentage of lesions in the corresponding HRCT scans.

**Table 2:** Clinical characteristics.

		AE-IPF (*n* = 48)	Stable IPF (*n* = 122)	Control (*n* = 70)
Gender	Male	48/48 (100%)	110/122 (90.2%)	63/70 (90%)
	Female	0/48	12/122 (9.8%)	7/70 (10%)
Age (yr)		65 ± 9	64 ± 8	59 ± 7
Environmental exposure^a^		8/48 (16.7%)^#^	26/122 (21.3%)^#^	6/70 (8.6%)
Surgical lung biopsy^b^		0	5	/
Smoking (%)		70.8^#^	68.9^#^	28.6
History of recent cold (%)		89.6^∗,#^	13.1^#^	0
Family history		0	1	0
1-year mortality		19/48 (39.5%)^∗,#^	35/122 (28.7%)	0
WBC (×10^9^/L)		9.01 ± 4.61^#^	8.06 ± 2.45^#^	6.01 ± 1.46
Neutrophils (%)		67.74±12.37^∗,#^	59.12 ± 9.55	60.17 ± 6.74
Lymphocytes (%)		22.62±10.38^∗,#^	29.58 ± 8.45^#^	32.46 ± 4.95
Monocytes (%)		6.45 ± 2.78^#^	7.30 ± 1.85^#^	5.87 ± 2.38
pH		7.43 ± 0.31^∗^	7.42 ± 0.26	NA
PaCO_2_ (mmHg)		37.92 ± 5.39	37.89 ± 5.46	NA
PaO_2_ (mmHg)		68.97 ± 16.45^∗^	80.47 ± 15.9	NA
SaO_2_ (%)		91.71 ± 6.98^∗^	94.83 ± 3.12	NA
FVC (% predicted)		59.35 ± 10.93^∗^	84.68 ± 22.62	NA
FEV1 (% predicted)		65.75 ± 10.78^∗^	84.89 ± 20.01	NA
FEV1/FVC		89.14 ± 8.01^∗^	80.65 ± 6.48	NA
TLC (% predicted)		66.24 ± 16.14	81.97 ± 19.26	NA
RV/TLC		43.06 ± 10.14	40.41 ± 5.68	NA
DLco (% predicted)		59.98 ± 19.50	62.88 ± 20.02	NA
HRCT scores		20.67 ± 5.98^∗^	12.27 ± 3.84	NA

^∗^ *p* < 0.05 vs. stable IPF; ^#^*p* < 0.05 vs. control. ^a^Patients were engaged in mining, painting, chemical manufacturing, teaching, carpentry, welding, warehouse managing, and farming. ^b^5 subjects were diagnosed with IPF by biopsy. Abbreviations: WBC: white blood cell; PaCO_2_: carbon dioxide partial pressure; PaO_2_: oxygen partial pressure; SaO_2_: oxygen saturation; FVC: forced vital capacity; FEV1: 1 second forced expiratory volume; TLC: total lung capacity; DLco: diffusion capacity for carbon monoxide; HRCT: high-resolution computed tomography; NA: not available.

**Table 3:** Specific IgM antibodies against 10 common pathogens (serum, number of positive cases, and positive rate).

IgM	AE-IPF (*n* = 41)	Stable IPF (*n* = 108)	Control (*n* = 70)
Mycoplasma	5, 12.2%	6, 5.6%	5, 7.1%
Legionella	3, 7.3%	5, 4.6%	0, 0%
Respiratory syncytial virus	2, 4.9%	4, 3.7%	0, 0%
Adenovirus	3, 7.3%	1, 0.9%	2, 2.9%
Influenza B virus	1, 2.4%	3, 2.8%	0, 0%
Cytomegalovirus	1, 2.4%	1, 0.9%	0, 0%
Chlamydia	0, 0%	1, 0.9%	0, 0%
Rickettsia	0, 0%	0, 0%	0, 0%
Parainfluenza Virus	0, 0%	0, 0%	1, 1.4%
Influenza A Virus	0, 0%	0, 0%	0, 0%
Total	15, 36.6%^∗^^#^	21, 19.4%	8, 11.4%

After chi-square test, ^∗^*p* < 0.05 vs. stable IPF; ^#^*p* < 0.05 vs. healthy controls.

**Table 4:** Pathogens in sputum of IPF patients.

Pathogens	Number of strains		
	AE-IPF (*n* = 48)	Stable IPF (*n* = 122)	Total (*n* = 170)
*Gram-negative bacteria*	9	25	34
*Klebsiella pneumoniae*	2	8	10
*Acinetobacter baumannii*	0	4	4
*Mycobacterium tuberculosis*	4	4	8
*Pseudomonas aeruginosa*	1	2	3
*Loffi Acinetobacter*	1	0	1
*Serratia marcescens*	0	1	1
*Enterobacter cloacae*	0	1	1
*Raoultella*	0	1	1
Other	1	4	5
*Gram-positive cocci*	0	4	4
Total no. of bacterial strain	9	29	38
Positive cases	9	26	35
Positive rate	9/48 (18.8%)	26/122 (21.3%)	35/170 (20.6%)

**Table 5:** Viruses in nasopharyngeal swabs of IPF patients.

Virus^a^	Number of viruses		
	AE-IPF (*n* = 30)	Stable IPF (*n* = 30)	Total (*n* = 60)
HHV	15	4	19
INF A	12	0	12
HRV	6	4	10
Other positive viruses^b^	7	9	16
Total positive viruses^c^	40	17	57
Positive cases	18	13	31
Positive rate	18/30 (60.0%)	13/30 (43.3%)	31/60 (51.7%)

^a^HHV: human herpesvirus; INF A: influenza virus A; HRV: human rhinovirus. ^b^Other positive viruses include AE-IPF: Andean potato latent virus; Mimivirus terra2; Tailam virus strain; halovirus; Emiliania huxleyi virus 86; oat dwarf virus; pepper mild mottle virus; stable IPF: Shamonda virus; Pandoravirus dulcis; pestivirus Giraffe-1; alfalfa mosaic virus; Megavirus chiliensis; murine osteosarcoma virus; Rous sarcoma virus; Y73 sarcoma virus; and hepatitis G virus. ^c^Some patients were positive for two kinds of virus

## Data Availability

All data used to support the findings of this study are available from the corresponding author upon request.

## Abbreviations

IPF: Idiopathic pulmonary fibrosis
UIP: Usual interstitial pneumonia
AE-IPF: Acute exacerbation of IPF
CT: Computed tomography
WBC: White blood cell
PaCO2: Carbon dioxide partial pressure
PaO2: Oxygen partial pressure
SaO2: Oxygen saturation
FVC: Forced vital capacity
FEV1: 1 second forced expiratory volume
TLC: Total lung capacity
DLco: Diffusion capacity for carbon monoxide
HRCT: High-resolution computed tomography.
